# What Do Therapist Defense Mechanisms Have to Do With Their Experience of Professional Self-Doubt and Vicarious Trauma During the COVID-19 Pandemic?

**DOI:** 10.3389/fpsyg.2021.647503

**Published:** 2021-07-30

**Authors:** Katie Aafjes-Van Doorn, Vera Békés, Xiaochen Luo, Tracy A. Prout, Leon Hoffman

**Affiliations:** ^1^Ferkauf Graduate School of Psychology, Yeshiva University, New York, NY, United States; ^2^Department of Counseling Psychology, Santa Clara University, Santa Clara, CA, United States; ^3^New York Psychoanalytic Society and Institute, New York, NY, United States

**Keywords:** defense mechanisms, therapist, COVID-19, vicarious trauma, professional self-doubt

## Abstract

This is the first study to examine psychotherapists' levels of defense mechanisms, their concurrent relationship with professional work-related stress (professional self-doubt and vicarious trauma), and how their levels of defense mechanisms predict the changes in these professional stresses over the course of 3 months since the start of the COVID-19 pandemic. Data from two online studies (Study 1; *N* = 105 and Study 2; *N* = 336), using two self-report measures of therapists' defense mechanisms (Defense Style Questionnaire-40 in Study 1 and Defense Mechanism Rating Scales Self-Report-30 in Study 2), are presented. Therapists reported higher levels of mature defense mechanisms, and lower levels of immature defense mechanisms, compared to published community and clinical populations assessed before and during the pandemic. Therapists' lower level of mature defense mechanisms and higher levels of neurotic and immature defense mechanisms were related to higher concurrent levels of vicarious trauma and professional doubt. Therapists who reported higher levels of mature defense mechanisms at 3-month follow-up showed less vicarious trauma and professional self-doubt at follow-up, after controlling for these professional stressors at baseline. Implications for clinical supervision and training are discussed. The context and professional challenges during the pandemic are unique and future replications of the results outside the pandemic context are warranted.

## Introduction

The concept of defense mechanisms has a long history in the field of psychology (Freud, 1894, 1936), in particular in the area of developmental psychology (e.g., Boldrini et al., 2020), psychopathology (Bond, 2004), and psychotherapy process and outcome research (e.g., Roy et al., 2009; Perry and Bond, 2012). Defense mechanisms, defined as automatic reactions to internal and external stressors or conflict, underlie a wide range of healthy and psychopathological phenomena (Perry, 2014). Individuals' choice of defense mechanisms is mostly involuntary, but the types of defense mechanisms used can lead to enormous differences in mental health and interpersonal effectiveness (Vaillant, 2020).

Defense mechanisms are categorized hierarchically based on their general level of adaptiveness (Perry, 1993; Perry and Bond, 2017). This hierarchy incorporates three overarching defense categories: (1) *Mature* defense mechanisms that include, for example, sublimation, altruism, anticipation, and humor; (2) *Neurotic* defense mechanisms that include intellectualization, undoing, isolation of affect, reaction formation, displacement, and repression; and (3) *Immature* defense mechanisms that include, for example, acting-out, splitting, projection, projective identification, idealization, devaluation, denial, passive-aggression, and help-rejecting complaining (Perry and Bond, 2017). In addition to these defense categories, many studies also report on overall defensive functioning (ODF); a summary variable consisting of the mean of each defense used, each weighted by its level. Lower ODF is generally associated with a greater number of symptoms, symptomatic behaviors and a wide variety of disorders (e.g., Drapeau et al., 2003; Kramer et al., 2013). Though all defense mechanisms are thought to protect the individual from anxiety, mature defense mechanisms do not threaten interpersonal relationships or distort reality as neurotic or immature defense mechanisms do.

Following the development of systematic assessment methods during the 1990s, recent studies have demonstrated a number of robust findings. There is ample research on therapists' judgments of patient defense mechanisms (e.g. Hendriksen et al., 2011), therapists' technique in response to patients' defense mechanisms (e.g., Winston et al., 1994; Siefert et al., 2006; Bhatia et al., 2016; Petraglia et al., 2017), accuracy of defense interpretation (Perry et al., 2012), and the role of patient's defense mechanisms and therapists' interventions in treatment alliance and treatment outcome (Despland et al., 2001). Improvement in the adaptiveness of defense mechanisms during psychotherapy is associated with greater adjustment and positive treatment outcome (e.g., Perry and Bond, 2012). Defense mechanisms are also a useful predictor of change in psychotherapy and have been shown to be malleable, with patients experiencing meaningful improvement in the type of defense mechanisms used after completion of psychotherapy (Babl et al., 2019). Recent findings demonstrated that defense mechanisms had a relevant impact on resilience to stressful life events, such as quarantining in response to the COVID-19 pandemic among community samples (DiGiuseppe et al., 2020a; Marazziti et al., 2020; Prout et al., 2020). However, with the exception of one study which showed that therapist trainees most frequently reported adaptive defense mechanisms (Adams and Riggs, 2008), little is known about therapists' own defensive functioning in normal professional circumstances or during the uniquely stressful time of the COVID-19 pandemic.

Although defense mechanisms generally become more salient when they are maladaptive, all human individuals use defense mechanisms in their daily lives (Cramer, 2008). Individuals tend to have certain default defense patterns that they use to manage distressing emotions and thoughts, but the use of specific defense mechanisms also depends on the circumstances, especially on the nature and level of distress (e.g., Perry et al., 2015; Békés et al., 2017). Stress has consistently been shown to be associated with the use of lower level defense mechanisms (e.g., Cramer, 2006; Perry et al., 2015), and those experiencing high levels of stress are likely to use more immature defense mechanisms than those experiencing less severe or no stress (Zimmerman et al., 2019).

Therapist factors play an important role in psychotherapy treatment outcomes. The fact that therapists differ significantly in their effectiveness, has mainly been examined by way of therapist effects, such as the interventions used, professional experience or training, and capacity for empathy (Constantino et al., 2017). It might also be important to consider other therapist factors on the personal qualities that are cross-situational and relatively constant across patients (i.e., inferred traits; see Beutler et al., 2004), such as the therapist's coping patterns, personality, attachment, and emotional wellbeing (Heinonen and Nissen-Lie, 2020). Indeed, interpersonal patterns that are characteristic to therapists showed the strongest evidence of a direct effect on the psychotherapy outcome (for a systematic review within the context of psychodynamic treatment outcomes, see Lingiardi et al., 2018). Similarly, Heinonen and Nissen-Lie, (2020), who systematically reviewed this literature across modalities, concluded that the most effective therapists are characterized by professionally cultivated, interpersonal capacities, which are likely rooted in their personal lives and attachment history.

The work of psychotherapy is known to be stressful for therapists, even in the best of times (Briggs and Munley, 2008) and working with patients triggers a range of emotional responses (Hayes et al., 2011). Many therapists also experience patient-contingent compassion fatigue and might subsequently experience burnout (Steel et al., 2015; O'Connor et al., 2018). It is thought that therapists' internal experiences and coping mechanisms may increase vulnerability to burnout (Simionato and Simpson, 2018). Therapists, for example, have a propensity to minimize their own vulnerability while continuing to expose themselves to excessive work pressures, to deny personal needs and emotions, and many are reluctant to set boundaries and ask for support. These factors appear to perpetuate the cycle of emotional exhaustion (Ledingham, 2015). Moreover, therapists themselves also experience their own emotional problems, such as anxiety and depression (Guy and Liaboe, 1986), and often pursue therapy for themselves (Orlinsky et al., 2011; Moe and Thimm, 2020).

The COVID-19 pandemic has posed a uniquely challenging situation for therapists (Aafjes-van Doorn et al., 2020a,b). This ongoing global crisis has had a significant negative impact on psychological distress and post-traumatic symptoms observed in both general and clinical populations (e.g., Prout et al., 2020; Tsamakis et al., 2020). When therapists empathically engage with these traumatized patients, they may experience a cumulative and deleterious effect through vicarious traumatization (McCann and Pearlman, 1990; Békés et al., 2020). From research on previous disasters, such as in Hurricane Katrina (Culver et al., 2011) or 9/11 (Boscarino et al., 2004), we know that the experience of vicarious trauma is especially impactful when therapists and patients are simultaneously experiencing a disaster. Moreover, in addition to managing widespread societal and health concerns, and often treating traumatized patients, therapists also suddenly had to adapt to providing online therapy during the current crisis (Békés et al., 2020). Transitioning from in-person to online therapy, without much time to access training or support, might make therapists less certain about their professional (clinical and technical) capacities and competencies (Aafjes-van Doorn et al., 2020a). Thus, given the generally increased stress during the time of COVID-19, when therapists are exposed to higher levels of patient distress and the sudden professional transition to an online format, they might revert to using lower level, less mature defense mechanisms.

Thus, therapists' experiences of professional self-doubt and vicarious trauma during the pandemic are likely to be affected by not only the external circumstances of the pandemic and by working online during this stressful time, but also their way of coping with these stresses. Examining therapists' use of defense mechanisms is especially important given that it might not only impact their experience of professional self-doubt and vicarious trauma and mental health in general, but also the quality of care they are able to provide to their patients.

Defense mechanisms serve a protective function in helping to maintain psychological integrity in the face of threat and are instrumental in determining ongoing adjustment to trauma (Punamaki et al., 2002). Although research examining specific defense mechanisms in relation to professional experiences, such as vicarious traumatization among therapists is lacking, vicarious traumatization has shown to be related to the level of defensive functioning among a sample of therapist trainees (Adams and Riggs, 2008). Also, recent studies have reported that therapists with healthy coping styles characterized by active, problem-focused strategies reported fewer PTSD symptoms, less vicarious traumatization, less negative affect, fewer disruptions in self-trust schemas, and less burnout than those with avoidant or emotion-focused coping styles (e.g., Schauben and Frazier, 1995).

This paper describes two studies—one cross sectional and one longitudinal. The aims of these studies were to address the following research questions: (1) What type of defense mechanisms did therapists use during the early days of the COVID-19 pandemic, as measured by two different defense mechanism assessment measures?; (2) How did therapists' defense mechanisms relate to their experiences of professional self-doubt and vicarious trauma when providing online therapy during the COVID-19 pandemic?; and (3) Were therapists' defense mechanisms related to their professional adaptation over the course of the first 3 months of the pandemic, as measured in Study 2?

We expected that therapists on average would report the use of relatively adaptive, mature defense mechanisms, and that higher levels of mature defense mechanisms would be related to less professional self-doubt and less vicarious traumatization experiences. We also hypothesized that therapists who used less adaptive defense mechanisms (i.e., relied more on defense mechanisms within the neurotic or immature defense categories) would show greater vulnerability to work-related stresses. We predicted that the type of defense mechanisms used would predict professional adaptation over time, in that therapists who use defense mechanisms in the mature defense category would experience more positive changes (reduction of professional-doubt and vicarious trauma experiences) over time compared to therapists who relied on neurotic or immature defense mechanisms.

## Methods

### Procedures

The two studies reported here represent two separate recruitment efforts of very similar online surveys. Both studies collected data during the COVID-19 pandemic. For Study 1, therapists were recruited between March 25 and May 17, 2020 (soon after the pandemic was declared by the World Health Organization and therapists had to suddenly transition to online therapy), via national and international professional listservs and individual contacts. For Study 2, therapists were recruited between April 11th and June 16th 2020 (in the early months of the pandemic outbreak in the United States) via additional professional email listservs for psychoanalysts and psychodynamically oriented clinicians, and social media outlets including Linkedin and Facebook.

In both these studies, interested therapists were directed to an online survey platform with additional information about the study. Therapists providing online therapy were eligible to participate. After providing consent, participants were directed to an online survey that included standardized scales in a fixed order, which took approximately 15 min to complete. The eligibility criteria, online consent procedures, and survey length were the same in both studies. Both studies were approved by [the local—omitted for peer review] Institutional Review Board. Other previous publications on this dataset have reported on the change in professional experiences over time (Aafjes-van Doorn et al., 2021).

### Measures

The online surveys in the two studies presented in this paper included the same individual items and standardized measures of professional self-doubt and vicarious trauma as well as additional scales unique to each study. Defense mechanisms were measured by different self-report measures in each study. For Study 1, therapists' defense mechanisms were assessed using the Defense Style Questionnaire-40 (DSQ-40; Andrews et al., 1993), because it was the most widely used defense measure in the literature. In Study 2, therapists' defense mechanisms were assessed using the newly developed Defense Mechanisms Rating Scale-Self Report-30 (DMRS-SR-30; DiGiuseppe et al., 2020a), because this promising newer measure, in contrast with the DSQ, provides an ODF metric. The DMRS-SR-30 has also been used in several large-scale COVID community studies and thus allows us to report on direct comparisons within the pandemic context. In both studies, the defense measure was administered once. In Study 1, a cross-sectional study, the defense measure (DSQ-40) was included. In Study 2, a longitudinal design, the defense measure (DMRS-SR-30) was part of the follow-up survey (to reduce the burden on participants who completed the lengthy baseline survey, the defense measure was only included in the shorter follow-up survey). Given the overlap in measures and similarity in study design, it was deemed most informative to report on these therapist-defense findings in conjunction in this manuscript, rather than in two separate manuscripts. We thus avoided piece-meal publications of these two studies.

#### Professional Self-Doubt

The Professional Self-Doubt scale (PSD; Nissen-Lie et al., 2017) is a nine-item scale derived from the larger Development of Psychotherapists Common Core Questionnaire (DPCCQ; Orlinsky et al., 1999). The PSD assesses therapists' level of uncertainty in their ability to be helpful for a patient by items such as feeling “*Afraid that you are doing more harm than good in treating a client*,” or “*Distressed by powerlessness to affect a patient's tragic life situation.”* Items are rated on a six-point Likert scale from 0 (never) to 5 (very often), with higher scores indicating more professional self-doubt. The PSD was assessed in Study 1 and at both timepoints in Study 2, Cronbach's α were 0.91 in Study 1 and 0.90 and 0.85 in Study 2.

#### Vicarious Trauma

The Vicarious Trauma Survey (VTS; Vrklevski and Franklin, 2008) is a self-report measure of subjective distress related to working with traumatized clients. The VTS includes eight items, from which the first two are screening questions about vicarious trauma exposure (e.g., “*My job involves exposure to distressing material and experiences*”), whereas the other six items ask about distress due to the exposure (e.g., “*It is hard to stay positive and optimistic given some of the things I encounter in my work*.”). In the present study only the six distress items were calculated without the two screening items (see Aparicio et al., 2013). Items are rated on a 7-point Likert scale from Strongly Disagree (1) to Strongly Agree (7), higher scores indicating more distress. The VTS has strong psychometric properties (Michalopoulos and Aparicio, 2012; Aparicio et al., 2013; Benuto et al., 2018). The PSD was assessed in Study 1 and at both timepoints in Study 2, Cronbach's α were 0.72 in Study 1 and 0.76 and 0.73 in Study 2.

#### Defense Mechanisms

##### Defense Style Questionnaire-40

In Study 1, defense mechanisms were assessed using the Defense Style Questionnaire-40 (DSQ-40; Andrews et al., 1993). The DSQ is the most widely used self-report measure of defense mechanisms (DiGiuseppe et al., 2020a). The DSQ is a 40-item self-report inventory that assesses individual defense mechanisms, structured into three defense categories: mature (8 items), neurotic (8 items), and immature (24 items). The DSQ uses a 9-point Likert scale from Strongly disagree (1) to Strongly agree (9). The DSQ-40 has strong psychometric properties, albeit the factor structure has been critiqued (e.g., Prout et al., 2018). Several researchers have reported difficulty in replicating the three-factor structure of the DSQ-40 (Trijsburg et al., 2000; Ruuttu et al., 2006; Prout et al., 2018; Tapp et al., 2018), to the extent that it is recommended not to use the DSQ subscales, without additional factor-analytic procedures on data obtained from the DSQ-40 (Wilkinson and Ritchie, 2015). The DSQ-40 was administered as part of the baseline survey. Cronbach's α for the DSQ total score was 0.91 in our study.

##### Defense Mechanisms Rating Scale-Self Report

In Study 2, defense mechanisms were assessed with the newly developed Defense Mechanisms Rating Scale-Self Report-30 (DMRS-SR-30; DiGiuseppe et al., 2020a). The DMRS-SR-30 is a self-report version of the observer-rated Defense Mechanisms Rating Scales (DMRS; Perry, 1990; Perry and Henry, 2004), both assess defense mechanisms across the hierarchy described in the DSM-IV (American Psychiatric Association, 1994). The DMRS-SR-30 uses a 5-point Likert scale ranging from Not at all (0) to Very often or Very much (4). The measure provides scores for three defense categories (Mature, Neurotic, Immature) based on 28 individual defense mechanisms, and a score for ODF. The psychometric properties of the DMRS-SR-30 show strong criteria and concurrent validity as well as convergent and divergent validity (DiGiuseppe et al., 2020a,b). The DMRS-SR-30 was administered as part of the follow-up survey. Cronbach's α in the present study was 0.85.

#### Demographics

Individual demographic items that were assessed in both studies included age, gender, race/ethnicity, highest degree, treatment orientation and setting, patient population, licensure, years of experience, number of patients, previous online therapy experience (yes/no), and previous training in online therapy (yes/no).

### Data Analysis

Standardized measures did not contain missing data because of the forced-choice logic of the online survey in Study 1 and Study 2. A small sample of participants (*N* = 26) completed both surveys for both Study 1 and Study 2; to maximize statistical power, these participants were included in the data analyses on aggregated means for both studies. We used the full sample of 105 psychotherapists in the analyses for Study 1 because all participants completed all standardized measures reported here. For Study 2, there was no missing data for the DMRS-SR-30 variables, but there was missing data for vicarious trauma and professional self-doubt. Therefore, we reported on the DMRS-SR-30 scores for all participants (*N* = 366), whereas the sample sizes of the correlations and regressions that included other variables were smaller (*N* = 178 for VTS, *N* = 169 for PSD). The completion rates of the VTS and PSD were lower because these measures were added midway through the data collection. The therapists who completed all measures did not differ significantly on therapist characteristics from those who did not complete the VTS and PSD in Study 2.

To answer the first research question, descriptive statistics of the three defense levels for both studies were reported. For the second research question, concurrent associations between defense categories and professional experiences for Study 1 and Study 2 were assessed using Pearson correlations. Because defense mechanisms were assessed only once in each study (at baseline in Study 1 and at follow-up in Study 2), the concurrent correlations were reported for the VTS and PSD data at the start of the pandemic (Study 1) and the VTS and PSD data at 3-month follow-up (Study 2).

For the third research question, the data from Study 2 was used to examine whether the use of defense mechanisms reported at the 3-month time point could predict reported professional experiences (VTS and PSD) at this same time point while controlling for experiences of professional self-doubt and vicarious trauma during the initial weeks of the pandemic, by using stepwise linear regression models. For completeness, paired sample *t*-tests to establish changes in vicarious trauma and professional self-doubt over time in Study 2 were reported. All the data were analyzed using IBM SPSS Statistics 25. Results for Study 1 and Study 2 will be reported sequentially.

## Results

### Study 1: Therapists' Defense Mechanisms During the Initial Weeks of the COVID-19 Pandemic

#### Therapist Characteristics (N = 105)

Therapists' mean age was 48.27 years old (*SD* = 15.78, range = 25–79). Most of the therapists were White (*N* = 90, 85.7%) and female (*N* = 92, 87.6%) and lived in the United States at the time of the survey (*N* = 93, 88.6%). Most therapists had received training as professional psychologists (*N* = 75, 71.4%). Most of them were licensed (*N* = 89, 84.8%) and relatively experienced (*N* = 69, 65.7% with more than 9 years of experience). Almost half of the participating therapists had no experience with online therapy prior to the pandemic (*N* = 47, 44.8%). See Table 1 for a more detailed description of the therapists' characteristics.

**Table 1:** Therapist characteristics in studies 1 and 2.

	**Study 1 (** ***N*** **=** **105)**		**Study 2 (** ***N*** **=** **336)**	
**Demographics**	***N***	**%**	***N***	***%***
**GENDER**				
Female	92	87.6	260	77.8
Male	13	12.4	76	22.2
**LOCATION CONTINENT**				
North America	94	89.5	294	87.5
Europe	9	8.6	34	10.1
South America	1	1.0	0	0
Australia and Oceania	1	1.0	2	0.6
Asia	0	0	3	0.9
Africa	0	0	1	0.3
ETHNICITY^*^				
White	90	85.7	281	83.6
Hispanic or Latino	6	5.7	10	3.0
Asian or Asian Indian	4	3.9	19	5.7
American Indian or Alaska Native	1	1.0	2	0.6
African American	0	0	0	0
Middle Eastern	0	0	5	1.5
Other/mixed	2	2.0	11	3.3
**PROFESSION**				
Psychologist	75	71.4	185	55.1
Social worker	14	13.3	42	12.5
Counsellor	8	7.6	19	5.7
Medical doctor	2	1.9	29	8.6
Other	9	8.6	69	20.5
WORK SETTING BEFORE THE PANDEMIC^*^				
Private Practice	75	71.4	263	78.3
Outpatient	28	26.7	58	17.3
Hospital	13	12.4	27	8.0
Other	7	7	8	2.4
THEORETICAL ORIENTATION^*^				
Psychodynamic	69	65.7	205	61.0
Integrative	51	48.6	124	36.9
CBT	44	41.9	87	25.9
Humanistic	28	26.7	56	16.7
Psychoanalytic	25	23.8	132	39.3
Systemic	17	16.2	41	12.2
Other	19	18.1	56	16.7
**Previous experience of providing online therapy**				
Yes	56	53.8	173	52.0
No	48	46.2	160	48.0
Previous training in providing online therapy^**^				
No	81	77.1	192	88.1
Yes	24	22.9	26	11.9

*Response categories are reported in order of prevalence in the samples*.

* *Multiple answers were possible per respondent*.

** *Due to a technical error, some responses on this item were missing*.

#### Therapists' Defense Mechanisms

Therapists reported a relatively high level of use of mature defense mechanisms (*M* = 6.18, *SD* = 0.87) as well as a relatively lower level of neurotic defense mechanisms (*M* = 4.20, *SD* = 0.88) and immature defense mechanisms (*M* = 2.69, *SD* = 0.81). Compared to the populations studied in the literature, therapists reported a significant lower level of immature defense use than community adults and neurotic patients in outpatient services (Sammallahti et al., 1996; Granieri et al., 2017). Therapists in Study 1 also reported higher use of mature defense mechanisms than the community sample reported by Granieri et al. (2017), but this difference was not significant when compared to another community sample or a neurotic patient sample. Therapists' reported neurotic defense use did not consistently differ from other samples (see Table 2).

**Table 2:** Independent samples *t*-tests comparing therapists' defense mechanisms (DSQ-40) in study 1 to other samples.

**Study**	**Sample**	***N***	**Mature defenses**	***t***	**Neurotic defenses**	***t***	**Immature defenses**	***t***
			***M* (*SD*)**		***M* (*SD*)**		***M* (*SD*)**	
Study 1	Therapists during pandemic	105	6.18 (0.87)		4.20 (0.88)		2.69 (0.81)	N/A
Granieri et al. (2017)	Community adults	328	4.82 (1.21)	**12.59**	4.36 (1.20)	−1.47	3.72 (1.06)	–**10.47**
				***p*** **<** **0.00001**		*p* = 0.14		***p*** **<** **0.00001**
Sammallahti et al. (1996)	Community adults	334	6.2 (1.4)	0.17	3.9 (1.3)	2.69	3.5 (1.0)	**8.43**
				*p* = 0.86		*p* = 0.007		***p*** ** < 0.00001**
Sammallahti et al. (1996)	Neurotic	53	5.7 (1.5)	2.15	4.2 (1.4)	0.0	4.8 (1.1)	**12.38**
	patients			*p* = 0.03		*p* > 0.99		***p*** ** < 0.00001**

*Independent sample t-tests were conducted between Study 1 and previous studies using DSQ in community or outpatient samples. Bonferroni correction was used to correct issues for multiple testing, with a significance level of p < 0.05/9 (0.006) for bolded items. All the comparisons were conducted with an assumption of unequal variance, given the differences in sample sizes*.

#### Concurrent Associations With Experience of Professional Self-Doubt and Vicarious Trauma

The descriptive statistics of experience of professional self-doubt and vicarious trauma and their Pearson correlations with the three defense categories are presented in Table 3. Immature defense mechanisms and neurotic defense mechanisms were positively related with professional self-doubt (*r* = 0.30, *p* = 0.002; *r* = 0.22, *p* = 0.03, respectively). Neurotic defense mechanisms were also positively associated with the level of experienced vicarious trauma (*r* = 0.23, *p* = 0.02).

**Table 3:** Pearson correlations between therapists' defense mechanisms (DSQ-40), professional self-doubt, and vicarious trauma in study 1 (*N* = 105).

**Variables**	***M* (*SD*)**	**1**	**2**	**3**	**4**	**5**
**DEFENSE MECHANISMS**						
1. Mature	6.18 (0.87)	–				
2. Neurotic	4.20 (0.88)	0.11	–			
3. Immature	2.69 (0.81)	0.02	0.51^**^	–		
**PROFESSIONAL EXPERIENCES**						
4. Professional self-doubt	2.48 (0.86)	−0.11	0.22^*^	0.30^**^	–	
5. Vicarious trauma	3.81 (1.16)	0.16	0.24^*^	0.17	0.47^**^	–

* *p < 0.05;*

** *p < 0.01*,

*** *p < 0.001*.

### Study 2: Therapists' Defense Mechanisms Three Months Into the COVID-19 Pandemic

#### Therapist Characteristics (N = 336)

Therapists' mean age in this sample was 50.05 years old (*SD* = 16.31, range = 22–84). Most of the participants were White (*n* = 291, 83.6%) and female (*n* = 260, 77.8%). Most of them lived in the United States (*N* = 278, 82.7%). The therapists were mostly trained as psychologists (*N* = 185, 55%), licensed (*N* = 283, 84.2%), and relatively experienced (*N* = 241, 71.5% with more than 9 years of experience). Most of them saw patients in private practice before the pandemic (*N* = 263, 78.3%) and worked with populations of adults (*N* =320, 95.2%) and adolescents (*N* =145, 43.2%). Approximately half of the participants had experiences providing online therapy prior to the pandemic *(N* =156, 46.5%). See Table 1 for a more detailed description of therapist characteristics.

#### Therapists' Defense Mechanisms

Similar to findings in Study 1, therapists reported high levels of mature defense mechanisms (*M* = 56.49, *SD* = 13.25) and relatively low levels of neurotic defense mechanisms (*M* = 21.04, *SD* = 6.92) and immature defense mechanisms (*M* = 10.12, *SD* = 5.35). The therapists' ODF (*M* = 5.71, *SD* = 0.50) indicated an average healthy (“healthy-neurotic,” Perry and Henry, 2004) functioning (Table 4). The ODF in our therapist sample was comparable to a community sample in Italy during the first week of lockdown (DiGiuseppe et al., 2020a,c), and significantly higher than an Italian sample of mostly students during the second month of the pandemic (DiGiuseppe et al., 2020c). Additionally, consistent with results in Study 1, therapists reported a significantly lower level of immature defense mechanisms than both community samples during pandemic (DiGiuseppe et al., 2020a,c), see also Table 4. Therapists reported a higher use of mature defense mechanisms than the community student sample and a higher use of neurotic defense mechanisms than the community adult sample (see Table 4).

**Table 4:** Independent samples *t*-tests comparing therapists' defense mechanisms (DMRS-SR-30) in study 2 to other samples.

**Study**	**Sample**	***N***	**Mature Defenses**	***t***	**Neurotic Defenses**	***t***	**Immature Defenses**	***t***	**ODF**	***t***
			***M* (*SD*)**		***M* (*SD*)**		***M* (*SD*)**			
Study 2	Therapists during pandemic	336	56.49 (13.25)		21.04 (6.92)		10.12 (5.35)		5.71 (0.50)	
DiGiuseppe et al. (2020a)	Young adults in Italy during pandemic	94	39.42 (10.08)	13.48	20.22 (5.73)	1.17	38.69 (9.18)	−28.84	4.91 (0.44)	**14.06**
				*p* < 0.00001		*p* = 0.24		*p* < 0.00001		***p*** **<** 0.00001^*^
DiGiuseppe et al. (2020c) (unpublished data)	Adults in Italy during pandemic	5,683	55.54 (19.99)	1.23	18.64 (10.65)	5.95	25.31 (14.94)	−43.06	5.58 (0.83)	2.84
				*p* = 0.22		*p* < 0.00001		*p* < 0.00001		*p* > 0.999

*Independent sample t-tests were conducted between Study 2 and previous studies using DMRS in community samples. Bonferroni correction was used to correct issues for multiple testing, with a significance level of p < 0.05/8 (0.0063) for bolded items*.

* *Other than this comparison, all the other comparisons were conducted with an assumption of unequal variance that is more conservative, given the differences in sample sizes. ODF = overall defensive functioning*.

While the majority of therapists (*N* = 218, 64.9%) reported healthy or superior functioning (ODF ≤ 5.5), a relatively large proportion (*N* = 93, 27.7 %) of therapists' defensive functioning fell into the range associated with neurotic character and symptoms disorders (5 ≤ ODF < 5.5). Finally, we identified a small percentage of therapists (*N* = 25, 7.4%) whose ODF fell into the lowest range, associated with personality disorders or acute depression (ODF < 5.0).

#### Concurrent Associations Between Defense Mechanisms and Experience of Professional Self-Doubt and Vicarious Trauma

The reported mean scores of the variables in Study 2, as well as the associations between experience of professional self-doubt and vicarious trauma and the three categories defense mechanisms, and ODF are presented in Table 5. Three months after the beginning of the pandemic, professional self-doubt and vicarious trauma experiences were negatively related with mature defense mechanisms (*r* = −0.40, *p* < 0.001, and *r* = −0.44, *p* < 0.001, respectively) and ODF (*r* = −0.37, *p* < 0.001, and *r* = −0.30, *p* < 0.001, respectively) while being positively related with neurotic defense mechanisms (*r* = 0.24, *p* = 0.003, and *r* = 0.25, *p* = 0.001, respectively).

**Table 5:** Pearson correlations between therapists' defense mechanisms (DMRS-SR-30), professional self-doubt, and vicarious trauma in study 2 (*N* = 336).

**Variables**	***M* (*SD*)**	**1**	**2**	**3**	**4**	**5**	**6**
**DEFENSE MECHANISMS**							
1. Mature	56.49 (13.25)	–					
2. Neurotic	21.04 (6.92)	−0.64^**^	–				
3. Immature	10.12 (5.35)	−0.62^**^	0.16^**^	–			
4. ODF	5.71 (0.50)	0.93^**^	−0.34^**^	−0.60^**^	–		
**PROFESSIONAL EXPERIENCES**							
5. Professional Self-Doubt	1.11 (0.68)	−0.40^**^	0.24^**^	0.31^**^	−0.37^**^	–	
6. Vicarious Trauma	3.97 (1.04)	−0.33^**^	0.25^**^	0.10	−0.30^**^	0.45^**^	–

* *p < 0.05;*

** *p < 0.01*,

*** *p < 0.001*.

#### Longitudinal Associations Between Defense Mechanisms and Experience of Professional Self-Doubt and Vicarious Trauma

Professional doubt significantly decreased over 3 months [*t*_(168)_ = 23.53, *p* < 0.001], whereas reported levels of vicarious trauma did not change significantly [*t*_(175)_ = 1.54, *p* = 0.127]. Given the significant correlations between defense mechanisms, professional self-doubt, and vicarious trauma, we conducted linear regression models to see if defense mechanisms (the total ODF score or the three defense categories) predicted these experiences of professional self-doubt and vicarious trauma at the 3-month time point, after controlling for professional self-doubt and vicarious trauma at the initial timepoint (i.e., early weeks of the pandemic). The results from the regression models, controlling for scores of professional self-doubt and vicarious trauma at the initial weeks of the pandemic, are presented in Table 6. After controlling the contribution of vicarious trauma at the early weeks of the pandemic, the ODF at the 3-month time point negatively predicted vicarious trauma (*B* = −0.36, *SE* = 0.13, *t* = −2.71, *p* = 0.01, Δ*R*^2^ = 0.03), with higher levels of defense functioning predicting lower levels of vicarious trauma. Similarly, the ODF at the 3-month time point also negatively predicted professional self-doubt (*B* = −0.35, *SE* = 0.09, *t* = −3.72, *p* < 0.001, Δ*R*^2^ = 0.05), after controlling for the significant contribution of professional self-doubt at the early weeks of the pandemic. Regarding the three categories of defense mechanisms (i.e., mature, neurotic, and immature), mature defense mechanisms at the 3-month measurement point negatively predicted vicarious trauma (*B* = −2.17, *SE* = 0.85, *t* = −2.56, *p* = 0.01, Δ*R*^2^ = 0.03) after controlling for the contribution of vicarious trauma, earlier in the pandemic, with higher use of mature defense mechanisms predicting lower levels of vicarious trauma. Similarly, after controlling for the significant contribution of professional self-doubt in the initial weeks of the pandemic, mature defense mechanisms after 3 months negatively predicted the experienced professional self-doubt at that same time (*B* = −1.16, *SE* = 0.58, *t* = −2.01, *p* = 0.046, Δ*R*^2^ = 0.06). In contrast, neither neurotic defense use, or immature defense use were associated with experiences of professional self-doubt and vicarious trauma in the regression model.

**Table 6:** Regression analyses of therapists' overall defensive functioning and defense mechanisms predicting change in therapists' experiences of professional self-doubt and vicarious trauma.

**Predictor variables**	**Coeff**.	***SE***	***95% CI***
**Vicarious Trauma Predicted by ODF**			
*Vicarious Trauma at the initial timepoint*	0.48^***^	0.06	0.37,0.59
*ODF*	−0.36^**^	0.13	−0.61, −0.10
	*R*^2^ = 0.35; *F*_(2, 173)_ = 46.67^***^		
**Professional Self-Doubt predicted by ODF**			
*Professional Self-Doubt at the initial timepoint*	0.38^***^	0.06	0.26,0.50
*ODF*	−0.35^***^	0.09	−0.53, −0.16
	*R*^2^ = 0.30; *F*_(2, 166)_ = 35.35^***^		
**Vicarious Trauma Predicted by Defense Levels**			
*Vicarious Trauma at the initial timepoint*	0.46^***^	0.06	0.35,0.58
*Defense Level*			
Mature	−2.17^*^	0.85	−3.85, −0.50
Neurotic	−0.94	1.30	−3.51, 1.63
Immature	−1.77	1.59	−4.90, 1.37
	*R*^2^ = 0.36; *F*_(4, 171)_ = 23.95^***^		
**Professional Self-Doubt Predicted by Defense Levels**			
*Professional Self-Doubt at the initial timepoint*	0.36^***^	0.06	0.24,0.49
*Defense Level*			
Mature	−1.18^*^	0.58	−2.33, −0.02
Neurotic	−0.35	0.91	−2.14, 1.44
Immature	1.13	1.09	−1.03, 3.28
	*R*^2^ = 0.31; *F*_(4, 164)_ = 18.17^***^		

*Coeff., Unstandardized coefficient; ODF, overall defense functioning. All variables in the regression were measured at follow-up (after controlling for Professional Self-Doubt and Vicarious Trauma at baseline)*.

* *p < 0.05;*

** *p < 0.01*,

*** *p < 0.001*.

## Discussion

To our knowledge, this is the first study to focus on defensive functioning among psychotherapists. Our aim was to assess defense mechanisms used by therapists during the early days and months of the pandemic, and to establish the concurrent relationship between use of defense mechanisms and experiences of professional self-doubt and vicarious trauma, as well as the relationship between defense mechanisms and change in these professional stressors over time. We reported on two recruitment efforts of two similar online surveys completed by therapists in the early days of the pandemic (Study 1) and 3 months into the pandemic (Study 2), using two different self-report measures of defense mechanisms, the DSQ-40 (Andrews et al., 1993) and the DMRS-SR-30 (DiGiuseppe et al., 2020b) respectively.

In response to the first research question, the results of both studies indicated that therapists reported relatively healthy levels of defense mechanisms during the pandemic. Therapists reported higher levels of mature defense mechanisms, and lower levels of immature defense mechanisms, compared to published community and clinical populations assessed before and during the pandemic. In Study 1, therapists on average reported higher levels of relatively adaptive, mature defense mechanisms and lower levels of neurotic and immature defense mechanisms, compared to published DSQ data on neurotic patient samples and community samples outside the pandemic (Sammallahti et al., 1996; Granieri et al., 2017). Similarly, in Study 2, therapists reported relatively healthy use of defense mechanisms, compared to other DMRS-SR-30 studies conducted during the pandemic. More specifically, therapists in Study 2 reported higher levels of ODF and higher levels of mature defense mechanisms than a small, Italian, young adult sample (DiGiuseppe et al., 2020a), but no significant difference with an Italian adult community sample (DiGiuseppe et al., 2020c). The therapists in Study 2 also reported lower levels of immature defense mechanisms than these young adult and adult community samples, and higher levels of neurotic defense mechanisms than the Italian community adults during the pandemic. Notably, defense levels among the therapists in both studies also varied greatly, ranging from low levels, usually associated with personality disorders and acute depression, through levels associated with neurotic character and symptom disorders, to healthy and superior level functioning (Perry and Henry, 2004).

In answer to our second research question, we found that therapists' lower level of mature defense mechanisms and higher levels of neurotic and immature defense mechanisms were related to higher concurrent levels of vicarious trauma and professional doubt. In other words, therapists who used less adaptive defense mechanisms showed greater vulnerability to professional self-doubt and vicarious trauma, whereas more adaptive defense mechanisms appeared to protect from these experiences. This relationship between defense mechanisms and professional stress was found in both studies, using two different defense mechanism rating scales. These results are in line with previous research showing that therapist trainees who used relatively mature defense mechanisms (as measured by the DSQ) reported fewer vicarious trauma symptoms (Adams and Riggs, 2008). This implies that by the use of mature, adaptive defense mechanisms, therapists may be able to manage the stress induced by the traumatic material they are exposed to in sessions, and thus decrease the likelihood of experiencing vicarious trauma. In contrast, the use of lower level, less adaptive defense mechanisms (neurotic, immature) increases the likelihood of more intense vicarious traumatization. More generally, this found association between neurotic and immature defense mechanisms and professional stress in therapists, fits with the literature on neurotic and immature defense mechanisms and psychological distress in the general population (related to anxiety in the general population; Mohamadpour, 2009; related to psychiatry residents' level of burnout; Hurşitoglu et al., 2019).

With regards to the third research question, therapists who reported higher levels of mature defense mechanisms at the 3-month follow-up in Study 2 showed less vicarious trauma and professional self-doubt at follow-up, after controlling for the level of these professional stresses at baseline. More specifically, higher ODF, as well as more mature defense mechanisms were associated with less professional self-doubt and vicarious trauma 3 months later, even when controlling for levels of these professional challenges during the initial weeks of the pandemic.

Previous studies have already shown that in patient and community populations, more adaptive defense mechanisms are related to better psychological functioning and less symptomatology (e.g., Perry and Bond, 2012), and studies during the pandemic supported these findings (e.g., DiGiuseppe et al., 2020a; Prout et al., 2020). Our results are in line with these results in that they indicate a positive relationship between the adaptiveness of therapists' defense mechanism and their experience of professional challenges like professional self-doubt and vicarious trauma. Our findings suggest that therapists' reported defense mechanisms may reflect their varying ability to cope with various professional stresses at the time of uncertainty and transition at the beginning of a global pandemic, as well as to adapt to the stresses over time.

When interpreting our results regarding therapists' defense mechanisms, and especially of those whose defense mechanisms fell into a lower range, it is important to keep in mind that data collection occurred during a global pandemic and in the midst of transitioning to provide online therapy. These particular circumstances inevitably color the picture. Our results may only reflect therapists' defensive functioning in the context of various personal and professional challenges. Given that stress is associated with the use of less mature defense mechanisms (Cramer, 2006; Perry et al., 2015), it is possible that therapists would have reported the use of more mature and less immature defense mechanisms outside the pandemic.

Keeping this in mind, there was a relatively high proportion of therapists in this sample whose ODF fell into a range often associated with neurotic and symptom disorders, and a small subsample of therapists whose ODF was at a low level, usually associated with personality disorders or acute depression. The prevalent use of maladaptive defense mechanisms is linked not only to symptomatology but, as our study has demonstrated, to experiences of professional self-doubt and vicarious trauma as well. The importance of therapists' defense mechanisms extends to the professional lives of therapists and the use of defense mechanisms likely has an impact on how they experience the practice of psychotherapy, and possibly the quality of support they provide to their patients.

Understanding characteristics of therapists that might explain their differences in outcome is a pressing task. Besides helping to better understand how psychotherapy works, knowledge on the characteristics of effective therapists could have other practical value. Insofar as adaptive defenses are trainable and defense-use is modifiable, training programs and supervision could be geared toward nurturing the use of more adaptive defenses. Also, merely being aware of these beneficial characteristics might help therapists monitor themselves in developing the qualities shown to improve outcomes via reflective and deliberate practice (Goldberg et al., 2016).

Similar to the advice given to patients, it might be important to provide professional and personal support to help therapists manage pandemic-related stress. Training on how to transition effectively to an online therapy format might be helpful in decreasing the overall stress of online work and may help increase therapists' professional confidence. Practicing self-care strategies and seeking out personal therapy, could improve therapists' ability to cope with the stress and trauma they experience during their online sessions during the pandemic and beyond.

Furthermore, therapists might benefit from identifying their own individual tendencies to use certain defense mechanisms when they experience stress, in and outside the professional context. Using an increased range of mature defense mechanisms might help build resilience and flexibility for adapting to future professional and societal challenges. These types of psychoeducation and personal-professional reflections could be integrated into graduate training curriculum and become part of supervision sessions, thereby helping to avoid high levels of professional self-doubt and vicarious traumatization during trainees' clinical practice and burnout later in their careers. Addressing therapists' use of defense mechanisms is especially important, given that therapists' professional stresses can also have a negative impact on the therapy process and ultimately treatment effectiveness (Sexton, 1999; Nissen-Lie et al., 2017).

## Limitations

Despite this unique contribution, several methodological limitations apply to our study. First, given that this study did not include a control group of online therapists assessed before the COVID-19 context, it is not clear if the therapist defense mechanisms and professional stresses reflect the intensity of the pandemic context, or if these associations would also emerge under normal professional and personal circumstances. It is therefore important to replicate this study outside of pandemic-times. Second, therapists' defense mechanisms were measured at one time point only (not repeated across measurement points), therefore it remains unclear if therapists revert to different more or less adaptive defense mechanisms over time. It is possible that many therapists become resilient and are able to tap into their pre-pandemic resources, whereas other therapists might experience accumulating stress over time (Aafjes-van Doorn et al., 2021), and revert to more maladaptive defense mechanisms as the pandemic continues. Third, a well-known limitation, common to all survey research, is that all variables were self-reported responses, which means that the relationship between these variables might have been spuriously inflated. Moreover, there is an inherent difficulty of assessing defense mechanisms through self-report measures (DiGiuseppe et al., 2020b) as the use of many defense mechanisms is relatively automatic and outside of awareness (Perry and Henry, 2004). However, both the DSQ-40 and the DMRS-SR-30 have been shown to have strong reliability and validity, and there is evidence that their results are comparable to observer-rated methods (DiGiuseppe et al., 2020b).

It also would have been helpful to have used the same defense measure for both samples of therapists, so that the samples could have combined into one large sample. Arguably, using two different defense measures has been valuable in itself, especially given that each measure has its own limitations. The DSQ, used in Study 1, has been widely used among patients and community samples, however, its face validity (e.g., Chabrol et al., 2005) and the factor structure has been criticized in the literature (e.g., Prout et al., 2018). The DMRS-SR-30 is newer and less widely used, but appears to be psychometrically stronger (e.g., DiGiuseppe et al., 2020a). Another limitation of the reported data is that in Study 2 only a subsample of DMRS-SR-30 completers were asked to complete the VTS and PSD. This was caused by the researcher's belated decision to add these measures to the survey, and does not represent missing data *per se*; nevertheless, it does limit the sample size of these correlations.

Regardless of the measure itself, statistically assessing defense mechanisms is intrinsically difficult. Studying defense mechanisms as they are manifested in internal experiences and behavior clouds the distinction between constructs (explanatory terms) and phenomena (empirical referents) (Mihalits and Codenotti, 2020).

Furthermore, although reflecting an international sample of therapists, the samples in the two studies are less diverse in other dimensions, such as race, educational level, and access to technology. Our recruitment efforts reflect convenience sampling, without equal subsamples of trainees, licensed clinicians, and those with or without training in online therapy. Further studies on larger and multicultural therapist samples are underway and might help to test if the prevalence of defense mechanisms, and their associations with therapists' experiences of professional self-doubt and vicarious trauma are generalizable to the therapist profession more widely.

## Conclusion

This study provides unique information about the therapists' use of defense mechanisms and experiences of professional self-doubt and vicarious trauma amidst a global pandemic. Therapists reported relatively adaptive levels of defense mechanisms, compared to published community and clinical populations assessed before and during the pandemic. During the initial weeks of the pandemic, as well as 3 months into the pandemic, adaptive defenses appeared as protective factors against experiencing vicarious trauma and professional doubt, whereas less adaptive (neurotic and immature) defenses appeared as risk factors of these professional stresses. Therapists who reported higher levels of adaptive defense mechanisms 3 months into the pandemic, showed reduced levels of vicarious trauma and professional self-doubt in these 3 months. Providing professional and personal support to therapists might help improve their psychological functioning and help manage their experiences of professional self-doubt and vicarious trauma, and ultimately help therapists to provide optimal care for their patients. Future replications of studies assessing therapists' defense mechanisms, as well as their relationship with professional stresses outside the pandemic context are warranted.

## Data Availability Statement

The data may be available by request. Requests to access these datasets should be directed to katie.aafjes@yu.edu.

## Ethics Statement

The studies involving human participants were reviewed and approved by Western IRB, Yeshiva University. The patients/participants provided their written informed consent to participate in this study.

## Author Contributions

KAVD and VB developed the survey study, conducted the study itself, as well as the data cleaning, data preparation, developed research questions, were responsible for the majority of the literature research, and write-up of the manuscript. XL contributed to the data preparation, data-analyses, and write up of the results. TP and LH contributed to the initial data collection, recruitment and survey design as well as several rounds of edits of the manuscripts. All authors contributed to the article and approved the submitted version.

## Conflict of Interest

The authors declare that the research was conducted in the absence of any commercial or financial relationships that could be construed as a potential conflict of interest.

## Publisher's Note

All claims expressed in this article are solely those of the authors and do not necessarily represent those of their affiliated organizations, or those of the publisher, the editors and the reviewers. Any product that may be evaluated in this article, or claim that may be made by its manufacturer, is not guaranteed or endorsed by the publisher.
